# Isotopes of ^210^Po and ^210^Pb in Hazel bolete (Leccinellum pseudoscabrum) – bioconcentration, distribution and related dose assessment

**DOI:** 10.1007/s11356-019-05376-8

**Published:** 2019-05-16

**Authors:** Karolina Szymańska, Dagmara Strumińska-Parulska, Jerzy Falandysz

**Affiliations:** 1 Toxicology and Radiation Protection Laboratory, Faculty of Chemistry, University of Gdańsk, Wita Stwosza 63, 80-308 Gdańsk, Poland; 2 Environmental Chemistry & Ecotoxicology Laboratory, Faculty of Chemistry, University of Gdańsk, Wita Stwosza 63, 80-308 Gdańsk, Poland; 3 Environmental and Computational Chemistry Group, School of Pharmaceutical Sciences, University of Cartagena, Zaragocilla Campus, Cartagena, Colombia; 4 Institute of Medicinal Plants, Yunnan Academy of Agricultural Sciences, Kunming, 650200 China

**Keywords:** ^210^Po, ^210^Pb, Mushrooms, Soil, Foraging, Effective radiation dose

## Abstract

The hazel bolete *Leccinellum pseudoscabrum* (Kallenb.) Mikšík 2017 specimens and beneath soil layer (0–10 cm) have been examined on the occasion of ^210^Po and ^210^Pb activity concentrations, the nuclide bioaccumulation potential by species and distribution in fruit bodies. Mushrooms and forest soils came from six geographically distant locations in the northern and central parts of Poland. The threat to humans from ^210^Po and ^210^Pb contained in mushrooms has been also assessed. The absolute values of the ^210^Po radioactivity, respectively, in caps and stems of fruit bodies were in the range 0.74 ± 0.06–8.59 ± 0.36 Bq kg^−1^ dry biomass and from 0.81 ± 0.06–8.23 ± 0.37 Bq kg^−1^ dry biomass, while the values of the ^210^Pb radioactivity in caps and stems were in the range 0.61 ± 0.04–6.33 ± 0.22 Bq kg^−1^ dry biomass and 0.83 ± 0.04–4.59 ± 0.24 Bq kg^−1^ dry biomass, respectively. A potential related effective dose assessment showed that mushrooms *L. pseudoscabrum* can contribute at 0.89–10.3 μSv kg^−1^ db from ^210^Po decay and 0.42–4.37 μSv kg^−1^ db from ^210^Pb decay.

## Introduction

The studied isotopes of ^210^Po (highly radiotoxic alpha emitter) and ^210^Pb (beta emitter) are widely present in the environment, both daughters of uranium ^238^U and exist as a naturally occurring radioactive material (NORM). Their half-lives are 138.38 days for ^210^Po and 22.3 years for ^210^Pb, while effective dose coefficients for ingestion, which measure a hazard of nuclear material given by its radiotoxicity arising from its radioactive characteristic, are 1.2 μSv Bq^−1^ and 0.69 μSv Bq^−1^, respectively (Persson and Holm 2011; ICRP 2012). Their occurrence in the atmosphere is a result of ^222^Rn decay diffusing from the ground when the short-lived ^222^Rn daughters (^218^Po → ^214^Pb → ^214^Bi → ^214^Po, ^210^Tl) quickly attach to airborne particles, decay as ^210^Pb → ^210^Bi → ^210^Po, and end up in the biosphere through dry and wet deposition (Skwarzec et al. 2001). Both isotopes, ^210^Po and ^210^Pb, are also observed in abiotic media (soil, sediment, water) in varying concentrations (Skwarzec et al. 2006, 2012; Strumińska-Parulska et al. 2010; Persson and Holm 2011). Food, and to a fewer extent aspirated aerosols, are the main sources of ^210^Po and ^210^Pb in humans (Pietrzak-Flis et al. 1997). Both nuclides are trace elements while their physical and chemical properties and radiotoxicity lead to their significant contribution to the overall radiation dose from foodstuffs. Hence, ^210^Po is classified as one of the most important nuclides among the naturally occurring radioisotopes for which humans are exposed (Pietrzak-Flis et al. 1997; Persson and Holm 2011). ^210^Po and ^210^Pb content in foodstuffs of plant origin and mushrooms depends on the geological structure of lithosphere, climate, and agronomic conditions (Persson and Holm 2011; Strumińska-Parulska and Olszewski 2018).

Wild growing mushrooms take part in soil weathering and decomposition of the organic substrate, and cycling the elements also constitute a source of food for forest animals and human (Falandysz and Treu 2017). Mycelium of many mushrooms well take-up from the soil substrata and bioconcentrate in flesh of fruit body various chemical elements including toxic mercury, cadmium, lead, and radioactive compounds that might rebound on health of human and animals, including game animals (Škrkal et al. 2015; Steinhauser and Saey 2016; Falandysz 2017). Among the persistent radionuclides, the most often studied in mushrooms is ^137^Cs, the artificial gamma emitter coming from the nuclear weapons use and testing and from nuclear power plant accidents, and the second is natural ^40^K (Mietelski et al. 2010; Steinhauser et al. 2014; Chatterjee et al. 2017; Cocchi et al. 2017; Prand-Stritzko and Steinhauser 2017; Tuo et al. 2017). Other radionuclides and some extremely toxic, mainly alpha emitters compounds, i.e., ^210^Po, ^210^Pb, ^234,238^U, ^228,230,232^Th, ^238^Pu, and ^239 + 240^Pu, which are much more laborious to study are with much less data in mushrooms (Mietelski et al. 2002; Vaaramaa et al. 2009; Guillén and Baeza 2014; Strumińska-Parulska et al. 2016, 2017; Szymańska et al. 2018).

Hazel bolete (*Leccinellum pseudoscabrum* (Kallenb.) Mikšík 2017) is a common, edible, and tasty mushroom, widespread in northern and central Europe. Despite its common name, hazel bolete is most often found close to hornbeam trees, although sometimes occurs with hazel trees.

The *L. pseudoscabrum* has no previous data on the radionuclides accumulated in mushrooms. This study intended to investigate the ^210^Po and ^210^Pb content, their bioconcentration potential by species and distribution in fruit bodies, and possible hazard of exposure to human consumers.

## Materials and methods

**Fig. 1:**
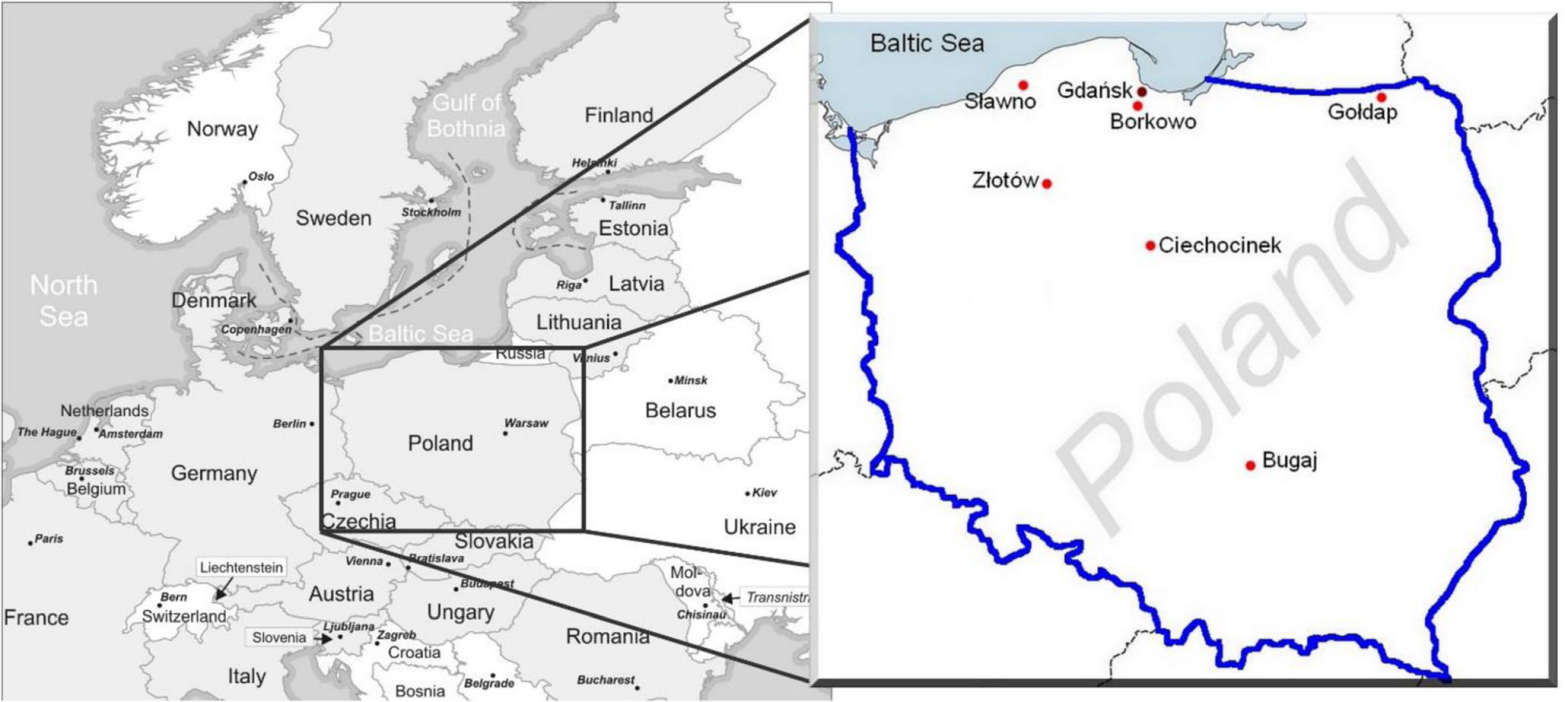
The map of hazel bolete (*L. pseudoscabrum*) fruit bodies and surface soil sampling locations

The hazel bolete (*Leccinellum pseudoscabrum* (Kallenb.) Mikšík 2017) fruit bodies and topsoil (0–10-cm layer) samples were collected from forests in the northern and central parts of Poland in 2000–2008. Mushrooms were collected in five places (Gołdap, Ciechocinek, Sławno, Złotów, and Borkowo) from the northern region, and one place (Bugaj) from the central region of the country, while topsoil samples from three locations (Sławno, Złotów, and Borkowo) (Fig. 1). Depending on the site, were collected from 3 to 18 composite samples of mushrooms (from 3 to 30 fruit bodies per a discrete sample). The places from the north of Poland, where the samples of *L. pseudoscabrum* were collected, have the acidic, leached brown soils—dystrophic autogenous soils formed from acidic rocks poor, containing 2–3% of organic matter, rich in potassium, calcium, and phosphorus. The Bugaj sampling location has podzolic soils with an organic matter content at 1.5–2% (Jadczyszyn et al. 2015).

All fruit body samples were separated for caps and stems, cut, and dried at 65 °C to constant weight. Further, the analyzed fungal materials were grinded up for a fine powder in a porcelain mortar. The topsoil samples were ridded of noticeable organisms and dried at 65 °C to stable mass as well. Next, the soil samples were sieved through a plastic sieve. Samples about 4–5 g of mushrooms and 2 g of soil substrate were taken and beforehand the radiochemical analysis, 9.5 mBq of ^209^Po was added as a yield tracer. All fungal materials prepared were digested using a solution of concentrated 65% HNO_3_ and further heated until acid has evaporated. According to soil samples, they were also digested using concentrated 65% HNO_3_ and HCl. Thanks to this we obtained, and were able to determine, the labile fraction of ^210^Po and ^210^Pb (not connected with the crystal structure of soil), available for living organisms. The dried residues of mushroom and soil samples were dissolved in 0.5 M HCl and a pinch of ascorbic acid was added. The polonium measurement discs were prepared by its autodeposition on pure 100% silver, the solution was transferred to PTFE vessels equipped with silver sheet bottom disc, and Po was autodeposited at 90 °C for 4 h. The activities of ^209^Po and ^210^Po were measured using an alpha spectrometer (Skwarzec 1997; Strumińska-Parulska et al. 2015, 2016). The ^210^Pb determination method was based on its indirect measurement via its daughter ^210^Po activity measurement (Strumińska-Parulska et al. 2015, 2016). All samples after the first ^210^Po deposition were evaporated and the dry residue was heated very strongly with 30% H_2_O_2_ added to remove potential polonium residues. Next, the samples were stored for 6 months to allow for ^210^Po increment from ^210^Pb. The deposition of ^210^Po on silver disc was repeated and the activities of ingrowing ^210^Po were measured in alpha spectrometer (Alpha Analyst S470, Canberra-Packard). The ^210^Po activity in analyzed samples was corrected for decay to the day of polonium deposition (time of separation ^210^Po from ^210^Pb). ^210^Pb activity at the time of sample collection was calculated using the simplified form of the Bateman equation (Skwarzec 1997). The chemical analysis efficiency of ^210^Po and ^210^Pb determination ranged 95–98%, while the results were given with standard deviation (SD) calculated for 95% confidence intervals.

An important question in chemical substances analysis of biota is their uptake and bioconcentration from soil, water, or air; bioaccumulation and biomagnification from the food net relationships; and distribution (fate) in a body. In the case of fungi (mushrooms), such information parameters could be described by the calculated values of bioconcentration factor (BCF) and, less frequently, the discrimination ratio (DR). The reduced assessment of soil-mushroom (through mycelium) radionuclide bioconcentration level could deliver the BCF (Eq. 1) (Gadd 2007; Strumińska-Parulska et al. 2016). Baeza and collaborators gave the discrimination ratio (DR) that could describe the different accumulations of the radionuclides in the cap + gills and stem (Eq. 2). As they wrote “a value of DR > 1 therefore indicates that the fruiting bodies accumulate this radionuclide in the cap+gills better than in the stem” (Baeza et al. 2006). The mushroom DR value might reflect the level of radionuclide uptake and its distribution into the caps and stems; however, the value of DR > 1 could also indicate the atmospheric fallout impact on radionuclides’ presence in the cap surface. In the end, a large variation of the DR values or its high values could be a result not only of the mushroom metabolism but also of the atmospheric deposition of ^210^Po and ^210^Pb onto the cap. It was not possible to differentiate these processes that impact on the DR value. The BCFs and DRs were calculated using presented formulas (Baeza et al. 2006; Strumińska-Parulska et al. 2016, 2017):

1 $$ {\mathrm{BCF}}_{\frac{\mathrm{whole}\ \mathrm{mushroom}}{\mathrm{soil}}}=\frac{\ \mathrm{Radionuclide}\ {\mathrm{activity}\ \mathrm{concentration}}_{\mathrm{whole}\ \mathrm{mushroom}}\kern0.50em \left[\mathrm{Bq}\ \mathrm{kg}-1\right]}{\mathrm{Radionuclide}\ {\mathrm{activity}\ \mathrm{concentration}}_{\mathrm{soil}}\kern0.50em \left[\mathrm{Bq}\ \mathrm{kg}-1\right]} $$

2 $$ {\mathrm{DR}}_{\frac{\mathrm{cap}}{\mathrm{stem}}}=\frac{\ \mathrm{Radionuclide}\ {\mathrm{activity}\ \mathrm{concentration}}_{\mathrm{cap}}\kern0.5em \left[\mathrm{Bq}\ \mathrm{kg}-1\right]}{\mathrm{Radionuclide}\ {\mathrm{activity}\ \mathrm{concentration}}_{\mathrm{stem}}\kern0.5em \left[\mathrm{Bq}\ \mathrm{kg}-1\right]} $$

## Results and discussion

### ^210^Po and ^210^Pb activity concentrations in hazel bolete and topsoil

The results of ^210^Po and ^210^Pb activity concentrations measured in *L. pseudoscabrum* and topsoil were given in Tables 1 and 2. Greater ^210^Po and ^210^Pb activity concentrations were noticed in the hazel bolete cap samples collected from the Borkowo place (Bo1): 8.59 ± 0.36 Bq ^210^Po kg^−1^ dry biomass (db) and 6.33 ± 0.22 Bq ^210^Pb kg^−1^ db than from other places. Obtained data on ^210^Po and ^210^Pb activity concentrations in caps and stems and knowledge on their biomass share within the whole fruit bodies enabled for calculations of the nuclide activity concentrations for entire mushrooms. Mushrooms collected from the location Bo1 (Borkowo) showed on the greatest activity concentration for both nuclides: 5.47 ± 1.04 Bq ^210^Po kg^−1^ db and 4.06 ± 0.63 Bq ^210^Pb kg^−1^ db than elsewhere. Also, forest topsoil from the Borkowo (Bo1) place was higher in ^210^Po and ^210^Pb, i.e., respectively, with 102 ± 2 Bq kg^−1^ dry mass (dm) and 100 ± 4 Bq kg^−1^ dm than elsewhere. The lowest ^210^Po and ^210^Pb activity concentrations were observed in hazel bolete caps from Sławno (Sł2): 0.74 ± 0.06 Bq ^210^Po kg^−1^ db and 0.63 ± 0.06 Bq ^210^Pb kg^−1^ db, while the lowest ^210^Po and ^210^Pb activity concentrations in topsoil were from the forests of the Złotów region, i.e., 18.8 ± 1.3 Bq ^210^Po kg^−1^ dm and 11.2 ± 0.4 Bq ^210^Pb kg^−1^ dm. The differences in ^210^Po and ^210^Pb content in soil, similarly to other elements, depended on its type, permeability, and fertility, and dark soil had greater sorption possibilities. It was noticed the organic soil contained three times more ^210^Po than mineral soil (Karanakura i in., 2000).

**Table 1:** The average values of ^210^Po activity concentrations for fruit bodies of hazel bolete (*L. pseudoscabrum*) and topsoil

Mushroom samples and sampling locations		^210^Po activity concentration (Bq kg^−1^ dry biomass)			
		Caps	Stems	Whole mushroom	Topsoil
Bugaj	Bu1	2.19 ± 0.14	1.95 ± 0.14	2.07 ± 0.88	n/a
	Bu2	2.03 ± 0.12	1.67 ± 0.14	1.87 ± 0.89	n/a
	Bu3	0.98 ± 0.08	1.03 ± 0.09	1.01 ± 0.57	n/a
	Bu4	2.00 ± 0.13	1.38 ± 0.11	1.69 ± 0.75	n/a
	Bu5	2.00 ± 0.14	1.51 ± 0.06	1.78 ± 0.69	n/a
Gołdap	Go1	2.53 ± 0.11	2.34 ± 0.12	2.44 ± 0.36	n/a
	Go2	3.11 ± 0.11	2.76 ± 0.15	3.02 ± 0.44	n/a
	Go3	3.38 ± 0.24	1.90 ± 0.13	2.45 ± 0.43	n/a
	Go4	2.69 ± 0.15	1.70 ± 0.08	2.12 ± 0.25	n/a
Ciechocinek	C1	4.06 ± 0.21	2.48 ± 0.16	3.28 ± 1.20	n/a
	C2	3.51 ± 0.21	3.07 ± 0.22	3.29 ± 1.29	n/a
	C3	3.75 ± 0.20	2.48 ± 0.15	3.13 ± 1.10	n/a
	C4	3.69 ± 0.03	2.73 ± 0.19	3.20 ± 1.09	n/a
	C5	2.06 ± 0.13	4.19 ± 0.18	3.14 ± 0.97	n/a
Sławno	Sł1	2.49 ± 0.13	2.31 ± 0.15	2.41 ± 0.82	25.3 ± 0.6
	Sł2	0.74 ± 0.06	2.02 ± 0.14	1.36 ± 0.65	31.5 ± 0.8
	Sł3	2.46 ± 0.17	1.80 ± 0.14	2.21 ± 0.60	29.8 ± 0.4
	Sł4	2.67 ± 0.14	3.20 ± 0.28	2.86 ± 0.93	28.9 ± 0.4
	Sł5	2.54 ± 0.24	1.85 ± 0.15	2.30 ± 1.05	37.8 ± 0.4
	Sł6	2.58 ± 0.23	2.80 ± 0.20	2.64 ± 1.08	28.5 ± 0.5
Złotów	Z1	2.67 ± 0.13	0.81 ± 0.06	1.91 ± 0.44	18.8 ± 1.3
Borkowo	Bo1	8.59 ± 0.36	1.05 ± 0.07	5.47 ± 1.04	102 ± 2
	Bo2	2.32 ± 0.17	1.87 ± 0.16	2.04 ± 0.45	45.8 ± 1.0
	Bo3	1.64 ± 0.08	8.23 ± 0.37	5.16 ± 1.11	48.1 ± 1.0

*n/a* not analyzed

**Table 2:** The average values of ^210^Pb activity concentrations for fruit bodies of hazel bolete (*L. pseudoscabrum*) and topsoil and ^210^Po/^210^Pb activity ratios

Mushroom samples and sampling locations		^210^Pb activity concentration (Bq kg^−1^ dry biomass)				^210^Po/^210^Pb activity ratio	
		Caps	Stems	Whole mushroom	Topsoil	Whole mushroom	Topsoil
Bugaj	Bu1	5.88 ± 0.44	1.10 ± 0.05	3.50 ± 2.01	n/a	0.59 ± 0.42	n/a
	Bu2	1.68 ± 0.10	0.83 ± 0.04	1.29 ± 0.56	n/a	1.45 ± 0.93	n/a
	Bu3	0.61 ± 0.04	0.83 ± 0.04	0.72 ± 0.26	n/a	1.40 ± 0.93	n/a
	Bu4	1.09 ± 0.08	0.78 ± 0.04	0.94 ± 0.39	n/a	1.80 ± 1.09	n/a
	Bu5	1.39 ± 0.09	0.89 ± 0.05	1.14 ± 0.45	n/a	1.54 ± 0.86	n/a
Gołdap	Go1	1.76 ± 0.09	1.51 ± 0.05	1.64 ± 0.25	n/a	1.49 ± 0.32	n/a
	Go2	1.70 ± 0.08	1.77 ± 0.08	1.72 ± 0.30	n/a	1.76 ± 0.40	n/a
	Go3	2.00 ± 0.12	0.99 ± 0.05	1.36 ± 0.19	n/a	1.79 ± 0.40	n/a
	Go4	1.73 ± 0.10	1.16 ± 0.07	1.40 ± 0.18	n/a	1.51 ± 0.26	n/a
Ciechocinek	C1	1.96 ± 0.04	1.51 ± 0.07	1.74 ± 0.34	n/a	1.89 ± 0.78	n/a
	C2	1.52 ± 0.07	1.41 ± 0.06	1.46 ± 0.38	n/a	2.25 ± 1.05	n/a
	C3	1.86 ± 0.09	1.56 ± 0.06	1.71 ± 0.50	n/a	1.83 ± 0.83	n/a
	C4	1.45 ± 0.10	1.07 ± 0.07	1.25 ± 0.53	n/a	2.57 ± 1.40	n/a
	C5	1.38 ± 0.06	2.53 ± 0.12	1.97 ± 0.63	n/a	1.60 ± 0.71	n/a
Sławno	Sł1	1.23 ± 0.09	1.63 ± 0.11	1.42 ± 0.59	25.1 ± 0.7	1.70 ± 0.91	2.21 ± 0.08
	Sł2	0.63 ± 0.06	1.66 ± 0.09	1.13 ± 0.50	38.1 ± 0.8	1.20 ± 0.78	1.81 ± 0.06
	Sł3	1.72 ± 0.15	1.32 ± 0.05	1.56 ± 0.49	30.0 ± 0.7	1.41 ± 0.58	2.12 ± 0.06
	Sł4	1.17 ± 0.11	0.94 ± 0.07	1.09 ± 0.50	30.2 ± 0.7	2.62 ± 1.48	2.12 ± 0.06
	Sł5	1.56 ± 0.12	1.59 ± 0.11	1.57 ± 0.53	40.1 ± 0.9	1.47 ± 0.84	1.95 ± 0.05
	Sł6	1.15 ± 0.08	1.51 ± 0.13	1.25 ± 0.43	30.6 ± 1.8	2.11 ± 1.13	1.91 ± 0.12
Złotów	Z1	1.95 ± 0.11	0.88 ± 0.09	1.52 ± 0.43	11.2 ± 0.4	1.26 ± 0.46	4.64 ± 0.34
Borkowo	Bo1	6.33 ± 0.22	0.85 ± 0.06	4.06 ± 0.63	100 ± 4	1.35 ± 0.33	1.27 ± 0.06
	Bo2	2.13 ± 0.16	1.17 ± 0.07	1.53 ± 0.28	106 ± 2	1.33 ± 0.38	0.52 ± 0.02
	Bo3	1.22 ± 0.11	4.59 ± 0.24	3.02 ± 0.77	59.2 ± 1.6	1.71 ± 0.57	1.24 ± 0.04

*n/a* not analyzed

**Fig. 2:**
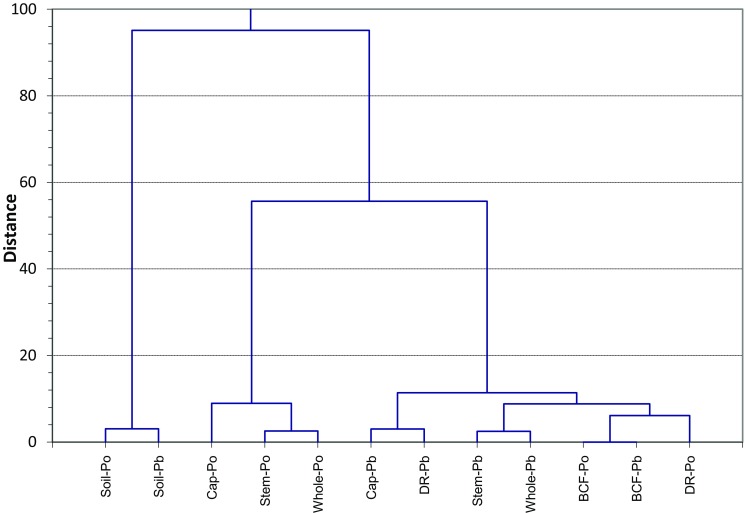
Cluster analysis (Ward’s method) based on the ^210^Po and ^210^Pb activity concentrations as well as BCF and DR values of analyzed mushrooms and the sampling places

**Fig. 3:**
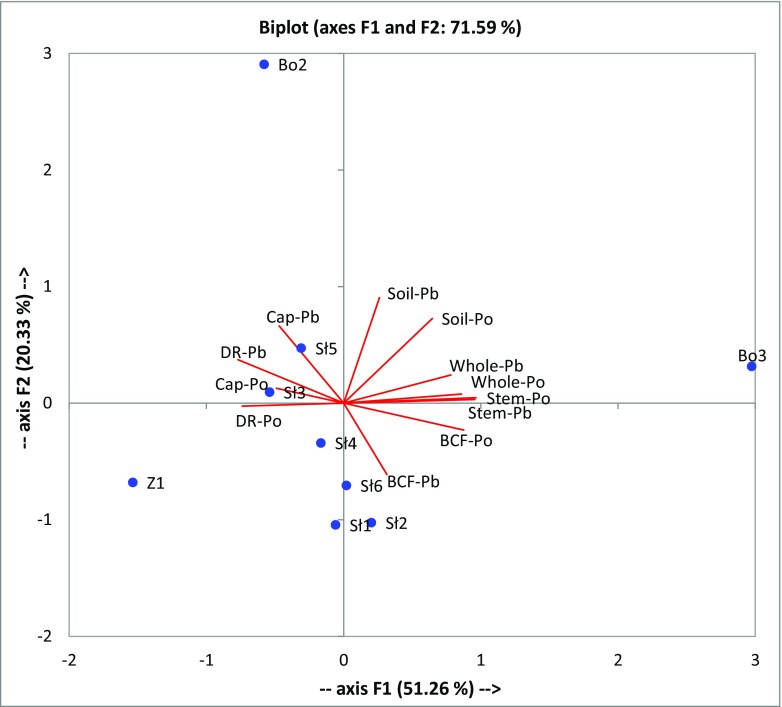
PCA analysis (no rotation) based on the ^210^Po and ^210^Pb activity concentrations as well as BCF and DR values of analyzed mushrooms and the sampling places (Sławno, Złotów, Borkowo)

The basic statistical analysis showed on significant differences in ^210^Po or ^210^Pb activity concentrations among the all mushrooms sampling locations (the *H* test (Kruskal-Wallis) *p* value 0.003 for ^210^Po and 0.0004 for ^210^Pb), while not between caps and stems of fruit bodies at every place sampled (the Mann-Whitney *U* test *p* value 0.08–1 for ^210^Po and 0.15–1 for ^210^Pb). In the case of the soil samples, the ^210^Po and ^210^Pb activity concentrations were diversified according to the sampling sites (*H* test *p* value was 0.06 for ^210^Po and 0.06 for ^210^Pb). The cluster analysis has been carried out also (Ward’s method) (Fig. 2). The resulting dendrogram shows that the clusters are linked at increasing levels of dissimilarity and with a strong connection between ^210^Po and ^210^Pb in soil samples. Also, the ^210^Po and ^210^Pb content of the whole mushrooms depended on their amount in stems (Fig. 2). The principal component analyses (PCA) has been carried out to demonstrate any possible spatial variations in the radionuclide activity concentrations between the Sławno, Złotów, and Borkowo places (Fig. 3). The analysis was based on the correlation matrix, and a fiducial significance level of *p* < 0.05 was chosen. The PCA data uncovered that 90.45% of information regarding the radionuclide compositional variability for three places could be described by three varifactors. The first varifactor (V1) described 51.26% of the total variance and loaded heavily on the positively correlated variables, describing ^210^Po and ^210^Pb in stems and whole mushrooms (loadings were 0.965, 0.956, 0.859, and 0.782, respectively). The second varifactor (V2) was loaded primarily by positively correlated ^210^Po and ^210^Pb in soil (loadings were 0.726. and 0.906, respectively) and explained 20.33% of the total variance (Fig. 3). Both analyses (cluster and PCA) showed that ^210^Po concentration in the soil samples related basically to ^210^Pb activity concentration in soil. The activity concentrations of ^210^Po and ^210^Pb in analyzed hazel bolete mushrooms were mostly similar to the other studies on genus *Leccinum* (e.g., foxy bolete, orange oak bolete, slate bolete, red-capped scaber) (Vaaramaa et al. 2009; Strumińska-Parulska et al. 2016; Szymańska et al. 2018), although much lower than determined in birch bolete (*Leccinum scabrum*) and orange birch bolete (*Leccinum versipelle*) collected in Norway (Gwynn et al. 2013).

Although Guillén and Baeza (2014) stated the most important feature in polonium accumulation in mushrooms was its species, our results confirmed previous observations (confirmed by inter-species studies)—the most important aspects in ^210^Po and ^210^Pb distribution are local climate and geological conditions, e.g., atmospheric fallout, geological structure, natural radioactivity (Vaaramaa et al. 2009; Gwynn et al. 2013; Strumińska-Parulska et al. 2016; Strumińska-Parulska and Olszewski 2018; Szymańska et al. 2018). Besides, in the forest area, the trees might decrease the influence of wet and dry deposition on soils and mushrooms (Kirchner and Daillant 1998; Strumińska-Parulska et al. 2017).

### ^210^Po and ^210^Pb BCFs and DRs

On the ground of ^210^Po and ^210^Pb activity concentrations in mushrooms and topsoil (Table 1), the values of BCF and DR were calculated (Table 3). The formula of soil-to-mushroom BCF (Eq. 1) gave information about the elements bioconcentration level. The values of BCF ranged 0.04–0.11 for ^210^Po and 0.01–0.14 for ^210^Pb (Table 2) and were lower when compared with the previously studied *Leccinum* spp*.* Low BCF values might be connected to soil substrate, namely brown acidic soil. Previous studies showed the highest BCF values were calculated for podzols while the lowest for brown soil (Strumińska-Parulska et al. 2016; Szymańska et al. 2018). Next, in the study, the ^210^Pb BCF values were lower when compared with ^210^Po BCF values (the Mann-Whitney *U* test *p* = 0.10), which confirmed the previous reports of stable lead (Pb) low bioconcentration (BCF < 1) capabilities by mushrooms (Jarzyńska et al. 2011; Strumińska-Parulska et al. 2016; Szymańska et al. 2018). Gadd (2007) has already noticed fungi may mobilize some metals, can methylate metalloids (e.g., selenium, tellurium, arsenic), or efficiently accumulate and immobilize some forms of metals (e.g., nickel, zinc, cadmium, lead). The *L. pseudoscabrum* due to the BCF values below unity (BCF < 1) is considered as excluder of ^210^Po and ^210^Pb, similarly to other mushrooms studied so far such as *Leccinum* sp. and parasol mushroom (*Macrolepiota procera*) (Strumińska-Parulska et al. 2016, 2017).

**Table 3:** The values of bioconcentration factor (BCF) and discrimination ratio (DR) for fruit bodies of hazel bolete (*L. pseudoscabrum*)

Mushroom samples and sampling locations		Bioconcentration factor (BCF)		Discrimination ratio (DR)	
		^210^Po	^210^Pb	^210^Po	^210^Pb
Bugaj	Bu1	n/a	n/a	1.14 ± 0.11	5.34 ± 0.48
	Bu2	n/a	n/a	1.42 ± 0.14	2.03 ± 0.16
	Bu3	n/a	n/a	0.96 ± 0.12	0.73 ± 0.06
	Bu4	n/a	n/a	1.44 ± 0.14	1.41 ± 0.12
	Bu5	n/a	n/a	1.33 ± 0.11	1.57 ± 0.13
Gołdap	Go1	n/a	n/a	1.12 ± 0.07	1.16 ± 0.07
	Go2	n/a	n/a	3.03 ± 0.20	0.96 ± 0.06
	Go3	n/a	n/a	1.03 ± 0.10	2.02 ± 0.16
	Go4	n/a	n/a	1.15 ± 0.09	1.50 ± 0.12
Ciechocinek	C1	n/a	n/a	1.69 ± 0.14	1.30 ± 0.06
	C2	n/a	n/a	1.17 ± 0.11	1.08 ± 0.06
	C3	n/a	n/a	1.57 ± 0.13	1.19 ± 0.08
	C4	n/a	n/a	1.47 ± 0.13	1.35 ± 0.13
	C5	n/a	n/a	0.48 ± 0.04	0.54 ± 0.04
Sławno	Sł1	0.10 ± 0.03	0.06 ± 0.02	1.19 ± 0.10	0.76 ± 0.07
	Sł2	0.04 ± 0.02	0.03 ± 0.01	0.38 ± 0.04	0.38 ± 0.04
	Sł3	0.07 ± 0.02	0.05 ± 0.02	2.19 ± 0.22	1.30 ± 0.12
	Sł4	0.10 ± 0.03	0.04 ± 0.02	1.53 ± 0.16	1.24 ± 0.15
	Sł5	0.06 ± 0.03	0.04 ± 0.01	2.68 ± 0.33	0.98 ± 0.10
	Sł6	0.09 ± 0.04	0.04 ± 0.01	2.25 ± 0.25	0.76 ± 0.08
Złotów	Z1	0.10 ± 0.02	0.14 ± 0.04	4.79 ± 0.40	2.21 ± 0.26
Borkowo	Bo1	0.05 ± 0.01	0.04 ± 0.01	11.6 ± 0.9	7.45 ± 0.62
	Bo2	0.04 ± 0.01	0.01 ± 0.005	0.74 ± 0.08	1.83 ± 0.17
	Bo3	0.11 ± 0.02	0.05 ± 0.01	0.17 ± 0.01	0.27 ± 0.03

*n/a* not available (soil not analyzed)

The estimated values of discrimination ratio (DR) (Eq. 2) might show the radionuclide differentiation in cap and stem and the impact of airborne particle fallout. The calculated DR values ranged 0.17–4.79 for ^210^Po and 0.27–5.34 for ^210^Pb (Table 2) and were similar to the previously studied *Leccinum sp.* mushrooms (described as “translocation factor”) (Strumińska-Parulska et al. 2016; Szymańska et al. 2018). The cluster analysis showed that ^210^Pb DR value was connected to ^210^Pb activity concentration in the cap, while ^210^Po DR depended on its BCF (Fig. 2).

### Annual effective radiation doses

**Fig. 4:**
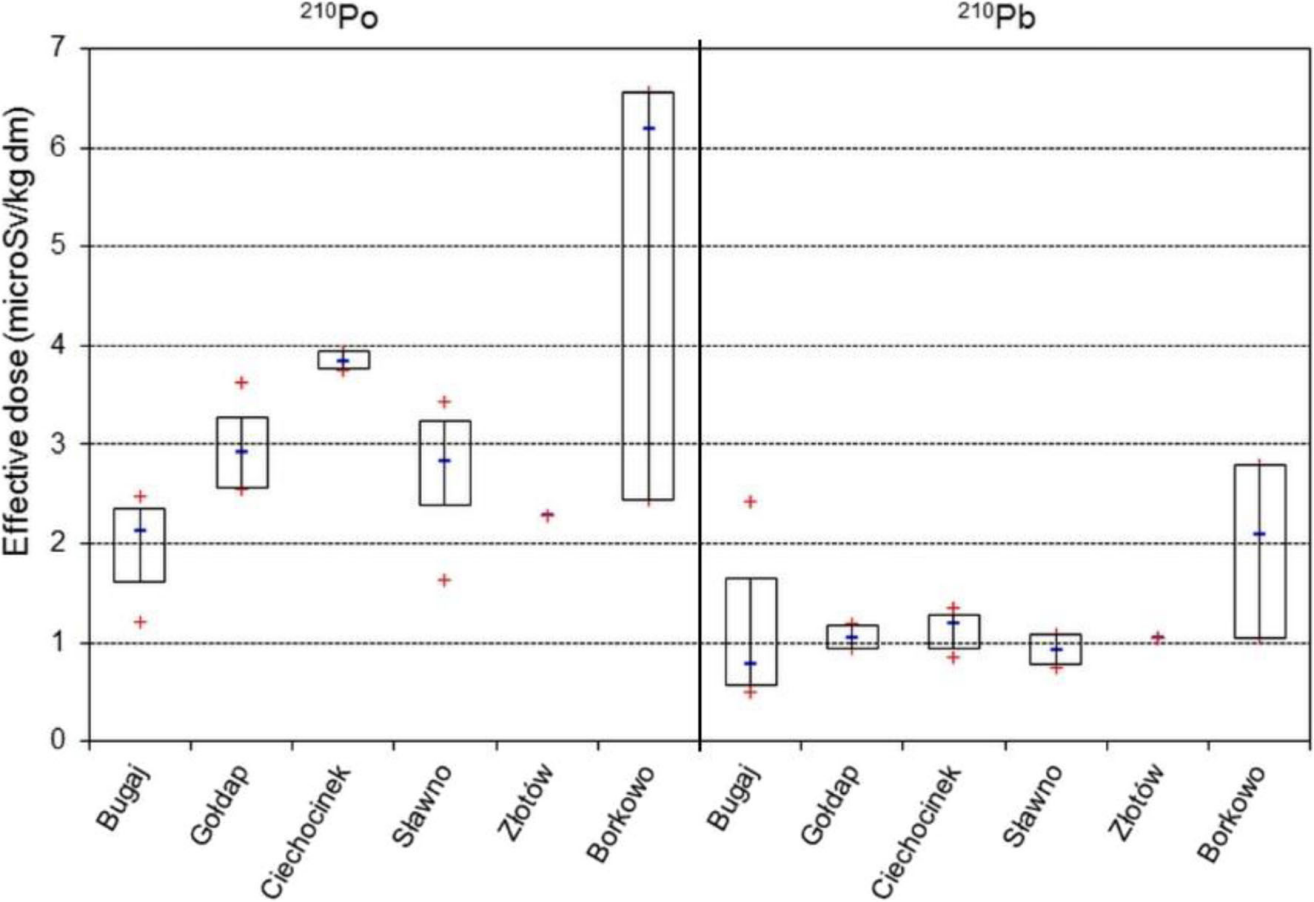
The assessed average values of the annual effective radiation dose from ^210^Po and ^210^Pb decay while ingested with hazel bolete (*L. pseudoscabrum*) mushrooms by adult

One of the important parts of food analysis is its safety evaluation. In order to estimate the potential radiotoxicity from ingested ^210^Po and ^210^Pb, the effective radiation doses for adult members of the public due to analyzed mushrooms consumption were calculated (Fig. 4) (ICRP 2012). According to the analyzed *L. pseudoscabrum*, the consumption of 1 kg of dried caps, stems, and/or whole fruiting bodies (equivalent to 10 kg of fresh mushrooms; assuming moisture content 90%) would give the effective radiation dose at 0.89–9.18 μSv from ^210^Po decay and 0.42–4.37 μSv from ^210^Pb decay. The annual effective dose from the total natural radiation in Poland was estimated at 2.1–2.6 mSv, while ^210^Po and ^210^Pb taken with food and water give 54 μSv (Pietrzak-Flis et al. 1997). Therefore, the evaluated effective doses from the intake of uncooked *L. pseudoscabrum* in volume equivalent to 1 kg of dried fruit bodies seemed to be low when compared with other domestic food products available in Poland (Strumińska-Parulska 2015; Strumińska-Parulska et al. 2016; Strumińska-Parulska and Olszewski 2018; Olszewski et al. 2019). Lead leaks at a high rate out of the fruit bodies in the course of the household treatment (e.g., blanching, pickling) or industrial processing (canning) (Drewnowska et al. 2017; Pankavec et al. 2019). Therefore, the intake rates of ^210^Pb from a mushroom meal (blanched-parboiled and further cooked or blanched/pickled) will result in the lower effective radiation dose than can be assessed for unprocessed produce. Basically, there is no data available on ^210^Po and ^210^Pb in cooked mushrooms or an impact of culinary processing.

## Conclusions

The studies indicated that ^210^Po and ^210^Pb are weakly bioconcentrated (BCF < 1) by hazel bolete (*L. pseudoscabrum*). The activity levels of ^210^Po and ^210^Pb in forest topsoil were low and together with low BCF values for both nuclides accounted for their low activity levels in fruit bodies of *L. pseudoscabrum*. Based on the data for raw mushrooms, consumption of *L. pseudoscabrum* could lead to effective doses at 0.89–9.18 μSv kg^−1^ db from ^210^Po decay and 0.42–4.37 μSv kg^−1^ db from ^210^Pb decay, which are safe from the radiological point of view.
